# The influence of weevil herbivory on leaf litter chemistry in dioecious willows

**DOI:** 10.1002/ece3.9626

**Published:** 2022-12-08

**Authors:** Joy M. Ramstack Hobbs, Iris J. Garthwaite, Logan Lancaster, Jordan A. Moffett‐Dobbs, Kelly Johnson, Nichole Criss, Victoria McConathy, C. Andrew James, Alex Gipe, Shannon M. Claeson, Carri J. LeRoy

**Affiliations:** ^1^ The Evergreen State College, Environmental Studies Program Olympia Washington USA; ^2^ St. Croix Watershed Research Station, Science Museum of Minnesota Marine on St. Croix Minnesota USA; ^3^ Center for Urban Waters University of Washington Tacoma Tacoma Washington USA; ^4^ Washington State Department of Ecology Lacey Washington USA; ^5^ USDA Forest Service, PNW Research Station Wenatchee Washington USA

**Keywords:** condensed tannins, dioecy, herbivory, mass spectrometry, Mt. St. Helens, Sitka willow

## Abstract

Leaf litter inputs can influence the structure and function of both terrestrial and adjacent aquatic ecosystems. Dioecy and herbivory are two factors that together have received little attention, yet have the potential to affect the quantity, quality, and timing of riparian litterfall, litter chemistry, and litter decomposition processes. Here, we explore litter chemistry differences for the dioecious Sitka willow (*Salix sitchensis* Sanson ex. Bong), which is establishing on primary successional habitats at Mount St. Helens (WA, USA) and is heavily infested with a stem‐boring weevil (*Cryptorhynchus lapathi*). Weevil‐attacked branches produced summer senesced litter that had significantly higher %N, lower C:N ratios, and lower condensed tannins than litter from branches that were unattacked by the weevil and senesced naturally in the autumn. Weevils more often attack female willows; however, these common litter chemicals did not significantly differ between males and females within the weevil‐attacked and ‐unattacked groups. High‐resolution mass spectrometry was used to isolate compounds in litter from 10 Sitka willow individuals with approximately 1500–1600 individual compounds isolated from each sample. There were differences between weevil‐attacked litter and green leaf samples, but at this level, there was no clustering of male and female samples. However, further exploration of the isolated compounds determined a suite of compounds present only in either males or females. These findings suggest some variation in more complex litter chemistry between the sexes, and that significant differences in weevil‐attacked litter chemistry, coupled with the shift in seasonality of litter inputs to streams, could significantly affect in‐stream ecological processes, such as decomposition and detritivore activity.

## Graphical abstract

In this paper, we explore the effects of dioecy and herbivory on leaf litter chemistry. The recently created headwater streams at Mt. St. Helens receive litter from the dioecious Sitka willow (*Salix sitchensis* Sanson ex. Bong), which is heavily infested with a stem‐boring weevil (*Cryptorhynchus lapathi*). This study suggests some variation in complex litter chemistry between the sexes and demonstrates that the weevil is altering not just the seasonality of litter inputs to these streams but also the chemical composition of that litter.

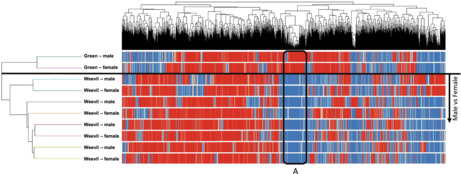

## 1 INTRODUCTION

Dioecy can influence plant physiology (Jones et al., 1999), herbivory (Boecklen et al., 1990), and mycorrhizal symbionts (Varga et al., 2013; Vega‐Frutis et al., 2015); however, the influence of dioecy on ecosystem processes like leaf litter decomposition has received little study (but see LeRoy, Ramstack Hobbs, et al., 2020), despite a meta‐analysis on how plant sex influences leaf chemistry (Cornelissen & Stiling, 2005). The meta‐analysis showed that female plants tend to exhibit significantly higher concentrations of secondary and other defense compounds, but that nutrient concentrations showed no significant difference between males and females (Cornelissen & Stiling, 2005). In a recent study, Yang et al. (2020) examined chemical differences in fresh leaves from male and female individuals of three *Salix* populations. In each population, they found no significant difference in condensed tannin concentrations between males and females but higher total phenolic content in the males. In two populations of *Salix suchowensis*, female willows had higher percent nitrogen than males. In a study of *Salix myrsinifolia*, Nybakken et al. (2012) found that females had higher concentrations of chlorogenic acids than males. This previous research provides a foundation from which we can explore additional litter chemistry differences between plant sexes.

In addition to dioecy, herbivory has the potential to influence ecosystem processes by altering both the quality and timing of leaf litter entering streams. Herbivores can increase summer “greenfall” (leaf material that is dropped fresh; Risley, 1986), induce “second‐flush” leaves of differing litter chemistry (Irons et al., 1991), change plant community composition (Sirotnak & Huntly, 2000), and alter the chemistry of herbivorized leaves by altering the translocation of nutrients, litter chemistry, and the timing of leaf abscission (Findlay et al., 1996). Summer greenfall due to herbivory tends to be higher in nitrogen than leaves that senesce in the autumn (Risley & Crossley, 1993). Greenfall from high wind, heavy rainfall events, or hurricanes is generally also higher in nitrogen than autumn litter (Fonte & Schowalter, 2004; Lodge et al., 1991; Lodge & McDowell, 1991). Fewer studies have explored the influences of herbivory on the “after‐life” effects on abscised litter chemistry (Findlay et al., 1996) and decomposition processes, and the results have been mixed, with some studies showing herbivory can increase intra‐specific litter quality and decomposition (Chapman et al., 2003), others showing decreases (Schweitzer et al., 2005), or both effects (LeRoy, Fischer, et al., 2020). These conflicting previous results make further exploration of herbivory influences on leaf litter, and possible interactions with plant sex, an important area of research.

Mount St. Helens (MSH, Washington, USA, Lawetlat'la in the Cowlitz language) is an ideal location to study the influences of interactions between dioecy and herbivory in a relatively simple landscape. In the years following the 1980 eruption, new stream channels have developed in the most disturbed zone, the Pumice Plain, from springs, seeps, and runoff from snowmelt (Blackman, 2014). Sitka willow (*Salix sitchensis* Sanson ex. Bong), a dioecious species, was one of the first species to establish on the Pumice Plain following the eruption (Wood & del Moral, 1988). The Sitka willows on MSH are heavily infested with a stem‐boring weevil (*Cryptorhynchus lapathi*), which causes branch dieback and summer litterfall (Che‐Castaldo et al., 2019). Both dioecy and herbivory have the potential to influence the quality and timing of Sitka willow litter entering the in‐stream detrital pool at MSH.

Previous work on the Pumice Plain has shown the potential importance of both dioecy and herbivory in this riparian‐aquatic ecosystem. A previous study found that female Sitka willows tend to grow closer to the stream edge and are more likely to be attacked by the stem‐boring weevil (LeRoy, Ramstack Hobbs, et al., 2020). Thus, females are contributing more litter to streams due to both proximity and herbivore attack. In addition, we found that summer weevil‐attacked litter from female willows was more recalcitrant (lower %N and higher C:N) and therefore functions as a resource over a longer period (LeRoy, Ramstack Hobbs, et al., 2020). Since the weevil causes branch dieback throughout the summer months, it therefore induces a shift in seasonality of litter inputs to streams (LeRoy, 2019; LeRoy, Ramstack Hobbs, et al., 2020). However, the extent of the differences in litter chemistry between weevil‐induced summer litter and naturally senesced autumn litter is not clear. Previous studies were limited in sample size to adequately address the influences of plant sex, herbivory, and their interaction. Finally, the chemical compounds examined by previous studies have been limited, and new analytical techniques, such as high‐resolution mass spectrometry (HRMS), could provide information on chemical‐specific differences associated with factors such as sex and herbivory, which may provide further insight into response mechanisms. HRMS instrumentation allows for the identification and differentiation of thousands of unique compounds in a sample, and subsequent data reduction and processing methods can provide information on differences between groups (Aydoğan, 2020; Di Ottavio et al., 2020). Resulting data can be further analyzed retrospectively to gain additional insight into specific chemical compositions or classes.

The purpose of this study was to analyze leaf litter from Sitka willow from across the Pumice Plain to determine the interactive influences of dioecy and herbivory (weevil attack) on litter chemistry. This was first accomplished by examining differences in traditional leaf litter chemistry measurements (C, N, and condensed tannins) among four litter types (male attacked, female attacked, male unattacked, and female unattacked). We also chose to analyze a small number of samples using HRMS to determine whether there were unique chemical occurrences in these litter types that could be further explored in future studies. Due to the expense of the HRMS technique, we use it here as a first‐step, exploratory tool to illustrate chemical differences among our Sitka willow sample groups. We hypothesized that our four litter types would (1) show differences in C, N, and condensed tannins and (2) have unique chemical fingerprints as determined by HRMS.

## 2 METHODS

### 2.1 Study site

**FIGURE 1:**
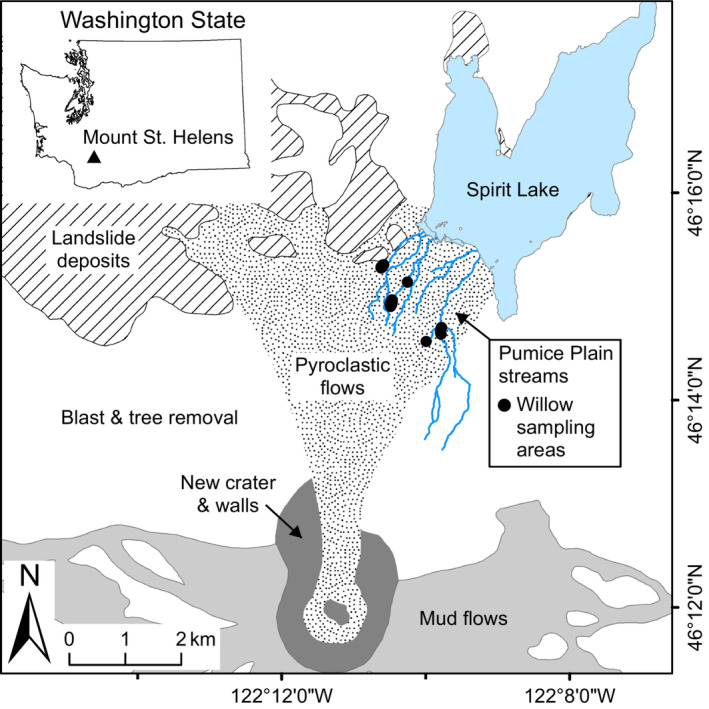
Map of study area. Inset shows the location of Mount St. Helens within Washington State (USA), larger map details the Pumice Plain. Black circles show locations of willow sampling areas.

When MSH erupted in 1980, over 600 km^2^ of the forested area was disrupted by a combination of pyroclastic flows, lahars, tephra fall, and hot air blasts (Lipman & Mullineaux, 1981). Besides the actual crater, the most disturbed zone was the area of pyroclastic flow, referred to as the Pumice Plain, which encompasses a 15 km^2^ area (Figure 1). The Pumice Plain was originally buried in over 100 m of pumice, ash, and sand in what was one of the largest terrestrial landslides in recorded history (2.8 km^2^ debris avalanche; Lipman & Mullineaux, 1981). The area was then hit with a hot lateral blast of flying rock debris and ultimately covered in 0.3 km^3^ of lava, in some places up to 40 m thick (Swanson & Major, 2005).

The eruption eradicated existing secondary temperate forests and streams that had been flowing north into Spirit Lake and the Toutle River drainage. In the years since 1980, a variety of new perennial and seasonal stream channels created five new watersheds on the Pumice Plain. Vegetation is slowly re‐establishing on the Pumice Plain, and several of the streams have developed significant riparian tree cover. Sitka willow and Sitka alder (*Alnus viridis* (Chaix) DC. ssp. *sinuata* (Regel) A. Löve & D. Löve) currently dominate the riparian habitats (del Moral & Jones, 2002). Willow shrubs are regularly attacked by an introduced stem‐boring weevil (*Cryptorhynchus lapathi*), which was first recorded on the Pumice Plain in 1989 (Che‐Castaldo et al., 2019).

### 2.2 Field methods

Individual Sitka willows (*N* = 349) were tagged as male or female in May of 2018 and 2019 when reproductive structures were present and sex determination was possible. Tagged individuals were revisited throughout the summers of both years and weevil damage was recorded. Litter for C, N, and condensed tannins (CT) analyses was collected in the summer and autumn of 2019. For male and female weevil‐attacked leaf litter, dry dead leaves were collected from tagged dead branches in June/July 2019. For male and female unattacked leaf litter, we collected nearly dry naturally abscising (yellowed, petiole lose) leaves from tagged willows in October 2019. Leaves to be analyzed with HRMS were slightly different to minimize seasonal differences and explore how herbivorized leaves differed from nonherbivorized leaves. For this smaller, exploratory aspect of the study, we collected weevil‐attacked leaves (dry dead leaves from dead branches) from a subset of the tagged individuals on 17 July 2018 and compared these samples with unattacked fresh (green) leaves collected on the same date.

### 2.3 Litter chemistry

Litter from individual male and female Sitka willows was collected in individual paper sacks in the field, freeze‐dried (Millrock Technology) in the laboratory, and ground to a homogeneous consistency using a KRUPS Type F203 grinder. Litter chemistry (%C, %N, C:N, and condensed tannins) was measured on samples from 45 individuals consisting of 9 unattacked females, 10 unattacked males, 16 attacked females, and 10 attacked males. To measure litter %C, %N, and C:N, subsamples (2 mg) of freeze‐dried, ground litter were weighed into 5 x 8 mm tin capsules and run on an elemental analyzer (2400 CHNS/O Series II System, Perkin Elmer). Soluble condensed tannins were extracted from additional subsamples (25 mg) of freeze‐dried, ground litter using 70% acetone and 10 mmol/L ascorbic acid. The butanol‐HCl method was used to determine soluble condensed tannin concentrations (Porter et al., 1986); standards were purified from Sitka willow following the methods in Hagerman and Butler (1989). Absorbance at 550 nm was measured on a spectrophotometer (SpectraMax 384; Molecular Devices).

### 2.4 High‐resolution mass spectrometry

Additional extracts (as for tannins) from a total of ten leaf litter samples (4 attacked males, 4 attacked females, 1 unattacked/green male, and 1 unattacked/green female) were further analyzed with quadrupole time of flight mass spectrometry (QTOF‐MS). For each sample, triplicate extracts were reduced to dryness at 50°C under a gentle stream of N_2_ and reconstituted in 1 ml LC/MS grade methanol (Fisher Scientific). Reconstituted samples were filtered through 13 mm diameter, 0.2 μm pore size PTFE syringe filters (Acrodisc 4423 T, Pall Corporation) and spiked with a set of 16 isotopically labeled internal standards.

Extracts were analyzed at the University of Washington Tacoma laboratories at the Center for Urban Waters (CUW) on an Agilent 1290 Infinity Ultra High‐Performance Liquid Chromatography (UHPLC) system coupled to an Agilent 6530 Quadrupole Time of Flight Mass Spectrometer (QTOF‐MS, Agilent Technologies). Analysis was performed in positive electron spray ionization with a full scan from 100 to 1700 m/z following the methods in Tian et al. (2020). Data reduction and analysis were performed as described by Tian et al. (2020) and Du et al. (2017). Briefly, peak identification and alignment across samples were performed with MassHunter Profinder (B08.00) and feature (where a “feature” is a unique chemical compound identified via HRMS) prioritization in Mass Profiler Professional (B13.00). Data reduction was performed by first, only retaining compounds with peak area > 5000, and present in all laboratory replicates. Next, the remaining compounds in each sample were compared with the compound set in solvent and method blanks and were only retained if they were present in samples at a peak area 5‐fold greater than blanks. The reduced compound set for each sample was used for suspect screening and to compare chemical occurrence by sample group. Suspect screening was performed by comparing the remaining compound set from each sample with two databases: an in‐house CUW database with retention time (RT), molecular formula, and measured mass for ~1100 compounds that have been run on CUW laboratory instrumentation, and the METLIN accurate mass compound database (https://metlin.scripps.edu/). Formula assignment and compound identification were performed in MassHunter ID Browser (B7.00) with compounds retained, which matched exact mass (<5 ppm), isotope pattern, and RT (<0.3 min) in the CUW database, or which matched exact mass and isotope pattern with assigned scores >85 of 100 via the METLIN database.

### 2.5 Statistical analyses

When analyzing traditional leaf chemistry data (C, N, C:N, and CT), variables were assessed for assumptions of normality and equality of variances, and ln‐transformations were used when necessary. To compare differences among the four litter types (male attacked, female attacked, male unattacked, and female unattacked) we used a two‐way ANOVA with explanatory variables of willow sex (male or female) and weevil status (attacked or unattacked), and their interaction (*n* = 45 plants). A post‐hoc Tukey test was run to determine significant differences in means among the four litter types. For the QTOF‐MS data sets, unique compounds between sample groups were identified by first, identifying compounds that were present in all samples from a given group (i.e., males or females) and then directly comparing the compound sets. The distributions of compound mass and % N in each compound were compared between male and female samples using permutative Kolmogorov–Smirnov tests. Analyses were performed using R version 3.6.0 (R Core Team, 2019) and with alpha = 0.05 unless otherwise stated. The overall similarity of compound occurrence patterns between sample groups was explored utilizing hierarchical clustering with reduced compound sets based on Euclidian distance calculations and Ward's linkages. In addition, nonmetric multidimensional scaling ordination with Euclidean distances was used to visualize differences among assemblages of chemical compounds, and multiresponse permutation procedures (MRPP) were used to compare assemblages among litter treatments (PC‐ord 6.0).

## 3 RESULTS

### 3.1 Carbon, nitrogen, and condensed tannins

**FIGURE 2:**
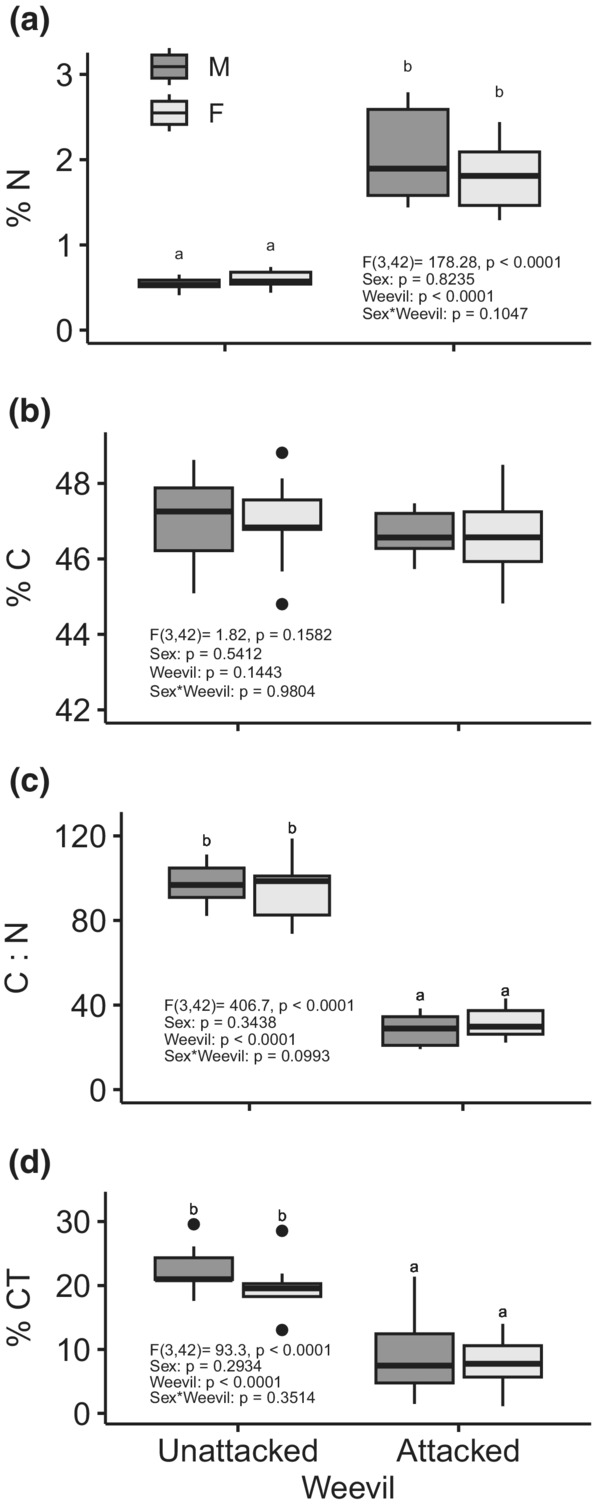
Differences in leaf litter chemistry between four groups (female unattacked, female weevil‐attacked, male unattacked, and male weevil‐attacked) for %N (a), %C (b), C:N (c), and % condensed tannins (%CT, d). Boxes denote the median, 25%, and 75% quartiles. Upper and lower bars indicate the minimum and maximum values, apart from outliers, which are represented by circles. Difference in the mean values was determined by Tukey tests and denoted by different lower case letters.

Standard leaf litter chemistry measurements (C, N, condensed tannins, CT) differed in response to herbivory but not dioecy (Figure 2). Weevil‐attacked litter (collected in summer) had significantly higher %N (*p* < .0001), lower C:N (*p* < .0001), and lower % CT (*p* < .0001) than leaves that were unattacked by the weevil and senesced naturally in the autumn (%N: *F*_(3,42)_ = 178.28, *p* < .001; C:N: *F*_(3,42)_ = 406.7, *p* < .001; %CT: *F*_(3,42)_ = 93.3, *p* < .001; Figure 2a‐d). Specifically, %N for male litter from weevil‐attacked plants was roughly 3.8 times higher than male litter from unattacked plants, and female weevil‐attacked litter was about 3 times higher than female unattacked litter in %N. Male unattacked litter was roughly 4 times higher in terms of C:N than weevil‐attacked litter, and female unattacked litter was 3.6 times higher than female weevil‐attacked litter in terms of C:N. Finally, male unattacked litter was roughly 3 times higher in terms of %CT than male weevil‐attacked litter, and female unattacked litter was 2.7 times higher than female weevil‐attacked litter in terms of condensed tannins. Male and female willows did not significantly differ within the attacked and unattacked groups for any of these variables (*p* > .05; Figure 2a‐d). In addition, there was no significant sex*weevil interaction for these variables (*p* > .05; Figure 2a‐d). Percent C did not differ by sex (*p* = .5412), weevil attack (*p* = .1443), or the sex*weevil interaction (*p* = .9804; *F*_(3,42)_ = 1.82, *p* = .1582; Figure 2a).

### 3.2 High‐resolution mass spectrometry

Results of QTOF‐MS were used to identify unique compounds in litter from 10 Sitka willow individuals collected on the same day during the summer: litter from dead branches on four weevil‐attacked male willows, litter from dead branches on four weevil‐attacked female willows, green leaves from one living branch of an unattacked male willow, and green leaves from one living branch of an unattacked female willow. Approximately 1500–1600 individual compounds were identified in each of the samples. This was run as an exploratory analysis with the objectives of identifying unique compounds across samples and sample types; therefore data reduction and analysis procedures were performed to achieve Level 4 (i.e., unequivocal molecular formula) confidence levels (Schymanski et al., 2014).

**FIGURE 3:**
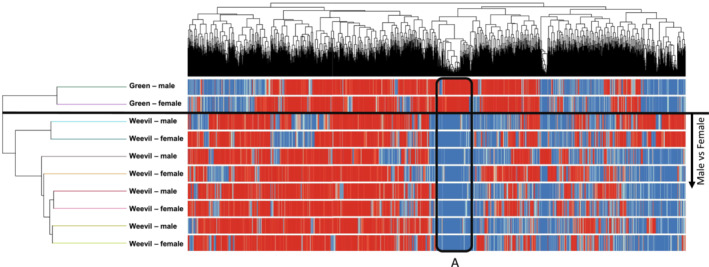
Results of hierarchical cluster analysis (Euclidian distance, Ward's linkage method) based on all log2‐transformed abundance of compounds present in all leaf samples identified with QTOF‐MS analysis. Red indicates a compound that was present in a given sample; blue indicates a compound that was absent from a given sample. The subclustering for male vs female for weevil‐attacked samples only (below black line) does not show distinct, sex‐based groupings. Box “A” showing compounds present in green samples but absent from all other samples is shown for illustrative purposes.

The results of a hierarchical cluster analysis indicated that the chemical profiles of the green leaf (unattacked) samples were distinct from the weevil‐attacked samples (Figure 3). There were no consistent differences between the chemical profiles in samples from male trees compared with the female trees. This comparison was extended through a MRPP with Euclidean distance measures, which showed a significantly different assemblage of compounds on green vs. weevil‐attacked leaf litter (A = 0.0784, *p* = .0211) but no difference between male and female litter (A = −0.009. *p* = .5792).

**FIGURE 4:**
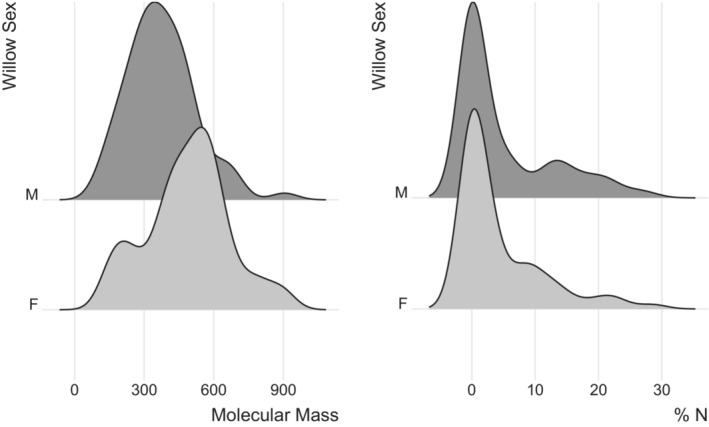
Plot showing the distribution of the masses and %N of 152 compounds present either only in male or only in female willow leaves.

Though there were no systematic differences between chemical profiles in the male and female leaf litter samples, there were compounds that were unique to either the male or female sample sets. A direct comparison of the male and female willows of the weevil‐attacked sample groups indicated that there were 152 compounds that were unique either to the males or females (Table 1). Seventy‐four compounds were unique to females and had an average of 4.26% N (±0.008%), while 78 were unique to males and had an average of 5.06% N (±0.009%), but distributions of compound‐specific %N were not different (*D* = 0.0977, *p* = .5667; Figure 4). The compounds unique to females also tended to have a higher mass, showing an average of 488.94 Da (±20.79) for females and an average of 375.82 Da ±16.65 for males (*D* = 0.3787, *p* < .0001; Figure 4).

**TABLE 1:** Table of compounds resulting from QTOF‐MS analysis that differentiate between weevil‐attacked leaf litter from male and female willows (*Salix sitchensis*).

Present only in females		Present only in males	
Mass	Formula	Mass	Formula
676.3681	C33 H56 O14	328.096	C18 H16 O6
441.2004	C21 H31 N O9	315.0882	C17 H15 O6
190.1359	C13 H18 O	370.3611	C27 H46
518.326	C26 H49 N O7 P	252.0644	C12 H12 O6
780.5563	C44 H79 N O8 P	316.2626	C18 H36 O4
441.2004	C21 H31 N O9	432.1073	C21 H20 O10
796.5141	C47 H72 O10	488.2174	C25 H32 N2 O8
554.201	C26 H34 O13	484.1329	C20 H24 N2 O12
602.3624	C38 H50 O6	357.2884	C20 H39 N O4
502.404	C32 H54 O4	357.2884	C20 H39 N O4
540.2202	C26 H36 O12	298.0858	C17 H14 O5
886.5595	C47 H83 O13 P	301.1093	C17 H17 O5
608.2661	C35 H36 N4 O6	520.1236	C24 H24 O13
528.4911	C36 H64 O2	464.2135	C17 H33 N6 O7 P
542.324	C28 H49 N O7 P	264.0683	C11 H12 N4 O2 S
870.5693	C55 H74 N4 O5	509.31	C24 H48 N O8 P
784.5885	C44 H83 N O8 P	222.162	C14 H22 O2
575.3712	C30 H49 N5 O6	394.3786	C26 H50 O2
514.1865	C27 H30 O10	107.0733	C7 H9 N
245.147	C9 H19 N5 O3	253.1676	C14 H23 N O3
469.1697	C25 H23 N7 O S	416.2389	C25 H33 F O4
602.3616	C38 H50 O6	268.0389	C15 H8 O5
432.1781	C23 H28 O8	412.1309	C15 H20 N6 O8
584.337	C34 H48 O8	348.3016	C23 H40 O2
574.4965	C37 H66 O4	389.2018	C14 H27 N7 O6
606.2501	C35 H34 N4 O6	606.1621	C28 H30 O15
224.1283	C12 H18 N O3	400.2076	C16 H28 N6 O6
472.2471	C24 H40 O7 S	222.162	C14 H22 O2
398.1263	C24 H18 N2 O4	342.278	C20 H38 O4
577.3862	C30 H51 N5 O6	545.2779	C26 H44 N O9 P
534.3719	C35 H50 O4	388.2109	C20 H28 N4 O4
561.3488	C29 H47 N5 O6	691.4076	C41 H57 N O8
689.344	C36 H51 N O12	296.2326	C18 H32 O3
430.3809	C29 H50 O2	622.1212	C27 H26 O17
912.715	C60 H96 O6	682.4608	C38 H67 O8 P
495.3714	C25 H54 N O6 P	298.2526	C18 H34 O3
442.17	C21 H30 O8 S	252.0653	C12 H12 O6
667.1918	C30 H35 O17	554.2163	C30 H34 O10
336.1812	C21 H24 N2 O2	472.2236	C30 H32 O5
564.29	C30 H44 O10	905.3209	C33 H55 N5 O24
654.1824	C29 H34 O17	498.2195	C18 H34 N4 O12
545.2968	C31 H39 N5 O4	648.4942	C39 H68 O7
610.3019	C37 H42 N2 O6	667.1933	C30 H35 O17
400.2471	C25 H36 O2 S	274.1796	C14 H26 O5
162.0528	C7 H14 S2	390.1949	C16 H30 N4 O5 S
516.1324	C18 H28 O17	159.0379	C6 H9 N O2 S
626.2796	C35 H38 N4 O7	307.0865	C10 H17 N3 O6 S
372.1876	C21 H28 N2 O2 S	142.135	C9 H18 O
274.1942	C18 H26 O2	337.163	C16 H23 N3 O5
582.2239	C35 H34 O8	332.2066	C14 H28 N4 O5
386.1234	C22 H18 N4 O S	383.3029	C22 H41 N O4
540.3408	C33 H48 O6	272.1045	C16 H16 O4
495.2666	C22 H42 N O9 P	376.3334	C25 H44 O2
152.1203	C10 H16 O	429.2373	C22 H31 N5 O4
194.0814	C8 H18 O S2	218.0801	C9 H14 O6
616.3768	C40 H48 N4 O2	434.0954	C19 H18 N2 O10
240.137	C14 H16 N4	194.0947	C11 H14 O3
362.16	C17 H22 N4 O5	152.121	C10 H16 O
450.1984	C16 H31 N6 O7 P	406.22	C19 H34 O9
290.189	C18 H26 O3	460.242	C22 H32 N6 O5
353.1989	C22 H27 N O3	253.0817	C9 H11 N5 O4
740.4366	C39 H64 O13	460.1394	C24 H20 N4 O6
389.2602	C20 H39 N O4 S	311.1636	C17 H26 Cl N O2
413.3133	C23 H43 N O5	349.179	C21 H23 N3 O2
376.2112	C18 H32 O8	192.1512	C13 H20 O
146.0366	C9 H6 O2	172.1107	C9 H16 O3
464.0963	C21 H20 O12	326.1352	C16 H22 O7
442.3804	C30 H50 O2	462.1535	C23 H26 O10
158.131	C9 H18 O2	301.159	C20 H19 N3
548.4504	C34 H60 O5	228.1723	C13 H24 O3
445.1843	C22 H27 N3 O7	434.2198	C23 H34 N2 O4 S
256.184	C18 H24 O	354.0966	C16 H18 O9
614.2389	C35 H34 Mg N4 O5	504.3189	C24 H40 N8 O4
191.1053	C10 H13 N3 O	198.1968	C13 H26 O
		476.1328	C23 H24 O11
		132.095	C10 H12
		488.2193	C30 H32 O6
		442.2736	C27 H38 O5

*Note*: Compounds on the left were present only in females, and compounds on the right were present only in males.

## 4 DISCUSSION

### 4.1 The role of dioecy

The effect of dioecy on leaf litter chemistry in this population of Sitka willow was not as straightforward as we had hypothesized. Instead, we found no significant differences between males and females in terms of C, N, or condensed tannins. The chemicals extracted by high‐resolution mass spectrometry were mostly similar across the samples we examined, and while there were consistent differences in males versus females, they were relatively small in number compared with the similarities. In addition, the male versus female difference was more pronounced for trees across similar conditions, with more than double the number of unique compounds identified when only the weevil‐attacked samples were included in the analysis. This exploratory analysis did not allow for the identification of the compounds isolated, so further work is needed to discover whether the differences in chemicals in male versus female willows are influential in terms of the fate or degradation of the leaf litter. Our previous work in this system with a smaller number of willows and litter collected in 2018 found that female weevil‐attacked litter had significantly lower %N and higher C:N (but no difference in %C or CT) and decomposed slower in streams than male weevil‐attacked litter (LeRoy, Ramstack Hobbs, et al., 2020). Based on other studies, which show the ability of different genotypes within species of *Salix* to accumulate heavy metals differentially (Mleczek et al., 2009), and many studies exploring within‐species variation in litter chemistry for other members of the Salicaceae family (LeRoy et al., 2007; Schweitzer et al., 2004, 2005), it is possible that randomly selected individuals may provide studies with small sample sizes spurious results that are not evident using larger sample sizes. It is also possible that there is significant seasonal (Rehill et al., 2006) or annual variation in litter chemistry that could have caused this discrepancy, and could be influencing the differences between weevil‐attacked and unattacked leaves. Future studies should compare chemical differences through time for both green leaves and leaf litter, and include experimental manipulations that lead to branch death and litterfall in the summer season without weevil attack.

Based on the differences in decomposition rates found between males and females in the 2018 study (LeRoy, Ramstack Hobbs, et al., 2020), it is possible that the reduced list of compounds found to distinguish males from females using HPMS analysis in this study may be of ecological importance. The 152 compounds that were unique to either males or females from the weevil‐attacked samples showed some consistency with what was found in our 2018 decomposition study in that the females tended to have lower %N and larger molecules (which could be more recalcitrant and slower to decompose). Future work should explore several of these novel compounds and their potential influences on leaf litter decomposition and aquatic detritivory.

### 4.2 The stem‐boring weevil as an ecosystem engineer

Previous work showed the stem‐boring weevil both selectively attacks female willows (Che‐Castaldo et al., 2019) and may cause a shift in the seasonality of litter inputs to the newly formed streams on the Pumice Plain (LeRoy, Ramstack Hobbs, et al., 2020). The results presented here suggest that the weevil is altering not just the seasonality of litter inputs to these streams but also the chemical composition of that litter. As with studies on the chemical composition of greenfall—litter that drops from trees prior to leaf abscission due to storms, hurricanes, or other herbivores (Feller, 2002; Fonte & Schowalter, 2004; Lodge et al., 1991; Lodge & McDowell, 1991; Risley & Crossley, 1993)—the weevil‐attacked litter was also found to be higher in nitrogen. Weevil‐attacked litter in this study also had lower C:N ratios and lower condensed tannin concentrations. These traits in combination mean that weevil‐induced summer litter differs greatly from leaves that normally senesce in the autumn and could mean that the streams are getting a larger pulse of N and labile litter in the summer, and that summer litter quality is likely higher and summer decomposition rates may also be faster.

However, the weevil‐attacked leaves in this study differ from what has typically been described as greenfall. One type of greenfall refers to green leaves falling during a storm (e.g., Lodge et al., 1991). QTOF‐MS analysis showed that the weevil‐induced litterfall in this study has a unique chemical signature compared with green leaves collected at the same time and location. These chemical changes may be induced by the herbivore attack or may be due to the inability of these willows to translocate resources prior to leaf abscision. In either case, the chemistry of the resultant litter differs from green leaves falling due to storms or other disturbances. Another type of greenfall referred to in the literature is used to describe green leaves falling when herbivore activity on the leaf causes petiole injury and resultant leaf detachment (e.g., Risley & Crossley, 1993). In this case, as with the stem‐boring weevil in this study, nutrients are unable to be resorbed from the leaves (as would occur with naturally senescing litter). However, in this study, the stem‐boring weevil causes premature mortality for the entire willow branch, meaning that the individual leaves have not been directly damaged by the herbivore. Future work should explore how this type of herbivory at the branch level induces changes in leaf‐level chemistry, differently than other types of herbivory.

### 4.3 Interactive effects of dioecy and herbivory

Although in this study, we did not see strong interactive effects between plant sex and herbivory on leaf litter chemistry, that does not necessarily mean these interactions are not important. Although the chemistry of male and female leaf litter responded similarly to herbivory by the stem‐boring weevil, spatial and temporal variation in of litter inputs are influenced by this interaction, with female willows both growing closer to stream edges (LeRoy, Fischer, et al., 2020; LeRoy, Ramstack Hobbs, et al., 2020) and more susceptible to weevil attack than males (Che‐Castaldo et al., 2019). These effects in combination mean that female willows interacting with a herbivore may have stronger influences on detrital dynamics in adjacent streams. Since many riparian species are dioecious (Freeman et al., 1980), it is possible that sex*herbivore interactions may have wide‐ranging influences on communities and ecosystem functions in both riparian and aquatic systems. Additionally, herbivores have been shown to alter terrestrial nutrient‐cycling and decomposition rates (Holland & Detling, 1990; Pastor et al., 1993; Sirotnak & Huntly, 2000; Stark et al., 2000; Wardle et al., 2002) and induce premature leaf abscission (Faeth et al., 1981; Hunter, 2001). Based on the vast range of herbivore effects, there are many possibilities for sex*herbivore interactions that remain underexplored.

### 4.4 Influences on headwater streams

Studies that have focused on herbivory‐inducing changes in leaf litter chemistry have primarily focused on the forest floor, and very few have considered litter inputs to streams (but see LeRoy, Ramstack Hobbs, et al., 2020; LeRoy, Fischer, et al., 2020). Our findings suggest that the significant differences in weevil‐attacked litter chemistry, coupled with the shift in seasonality in inputs to streams, could have significant effects on in‐stream ecological processes, such as decomposition and microbial and macroinvertebrate interactions. Future studies examining decomposition rates between these litter types in streams on the Pumice Plain could address the hypothesis that weevil‐attacked litter may be more labile and shift litter inputs earlier and therefore serve as a resource in the streams for a longer period. During mid‐summer, stream flow on the Pumice Plain is generally more stable than during spring or fall. Therefore, the shift in the seasonality of organic matter inputs caused by the weevil means that attacked litter inputs are occurring at a time of greater hydrologic stability when litter is more likely to be a stable resource for macroinvertebrates (less likely to be flushed downstream by spring and fall floods, and less likely to be entering just as the streams go dry in the late summer and fall). Weevil‐influenced litter inputs also coincide with reproductive inputs like male and female catkins, which have been shown to have lower concentrations of condensed tannins and host different assemblages of macroinvertebrates (Garthwaite et al., 2021). In nutrient‐limited early successional streams, interactions among dioecious riparian plants, herbivores, and resultant changes to leaf litter chemistry may have broad influences on allochthonous litter inputs, community development, and in‐stream ecosystem functions.

## AUTHOR CONTRIBUTIONS

**Joy M. Ramstack Hobbs:** Conceptualization (lead); data curation (equal); formal analysis (equal); investigation (equal); methodology (equal); project administration (equal); supervision (equal); visualization (equal); writing – original draft (lead); writing – review and editing (lead). **Iris J. Garthwaite:** Formal analysis (supporting); investigation (supporting); writing – review and editing (supporting). **Logan Lancaster:** Formal analysis (supporting); investigation (supporting); writing – review and editing (supporting). **Jordan A. Moffett‐Dobbs:** Formal analysis (supporting); investigation (supporting); writing – review and editing (supporting). **Kelly Johnson:** Formal analysis (supporting); investigation (supporting); writing – original draft (supporting); writing – review and editing (supporting). **Nichole Criss:** Formal analysis (supporting); investigation (supporting); writing – review and editing (supporting). **Victoria McConathy:** Formal analysis (supporting); investigation (supporting); writing – review and editing (supporting). **C. Andrew James:** Conceptualization (supporting); formal analysis (equal); investigation (equal); methodology (equal); validation (equal); visualization (equal); writing – original draft (supporting); writing – review and editing (supporting). **Alex Gipe:** Formal analysis (equal); investigation (supporting); methodology (supporting); writing – original draft (supporting); writing – review and editing (supporting). **Shannon Claeson:** Conceptualization (supporting); investigation (equal); project administration (equal); supervision (supporting); writing – original draft (supporting); writing – review and editing (supporting). **Carri LeRoy:** Conceptualization (supporting); data curation (equal); formal analysis (equal); funding acquisition (lead); investigation (equal); methodology (equal); project administration (lead); resources (lead); supervision (equal); validation (equal); visualization (equal); writing – original draft (supporting); writing – review and editing (supporting).

## CONFLICT OF INTEREST

The authors do not have any conflicts of interest.

## Data Availability

Upon acceptance of the manuscript, data will be deposited in GitHub at: https://github.com/carrileroy/Hobbs_et_al.
